# Concurrent stricturoplasty with lumen-apposing metal stents for gastrojejunal strictures after Roux-en-Y gastric bypass

**DOI:** 10.1007/s00464-026-12844-z

**Published:** 2026-04-29

**Authors:** Xinlei Zhu, Valentin Mocanu, Pattharasai Kachornvitaya, Mélissa V. Wills, Andrew Strong, Salvador Navarrete, Yung Lee, Juan S. Barajas-Gamboa, Ricard Corcelles, Jerry Dang, Matthew Kroh

**Affiliations:** 1 Digestive Disease Institute, Cleveland Clinic, Cleveland Clinic Lerner College of Medicine of Case Western Reserve University, 9500 Euclid Avenue, Cleveland, OH 44195 USA; 2 Cleveland Clinic Lerner College of Medicine of Case Western Reserve University, Cleveland, OH USA; 3 Department of General Surgery, Digestive Disease Institute, Cleveland Clinic Abu Dhabi, Abu Dhabi, UAE

**Keywords:** Metabolic and bariatric surgery, Sleeve gastrectomy, Roux-en-Y gastric bypass, Lumen-apposing metal stents, Gastrojejunal stricture

## Abstract

**Background:**

Gastrojejunal (GJ) anastomotic strictures are a challenging complication following Roux-en-Y gastric bypass (RYGB) and may be refractory to conventional balloon dilation. Lumen-apposing metal stents (LAMS) provide anchored, wide-caliber support that may treat strictures, yet long-term outcomes remain variable, and stent migration is a concern. This study evaluates the clinical effectiveness and safety of LAMS with or without concurrent endoscopic stricturoplasty in patients with GJ strictures.

**Methods:**

We conducted a single-center retrospective cohort study of patients with GJ strictures who underwent LAMS placement between January 2020 and May 2025. Patients were stratified by whether concurrent endoscopic stricturoplasty was performed at the time of stent placement. Clinical success was defined as symptom resolution without need for reintervention. The primary outcome was long-term clinical success (> 90 days). Secondary outcomes included short-term clinical success (≤ 30 days), stent dwell time, procedure duration, stent migration, and adverse events.

**Results:**

Twenty-five patients were included (mean age 46.8 ± 12.9 years; 76% female), with 7 (28%) undergoing concurrent stricturoplasty. The overall cohort short-term and long-term clinical success rates were 88.0% and 64.0% respectively. Long-term success was 71.4% in the stricturoplasty group vs. 61.1% in the LAMS-only group (p = 0.985). Stent migration occurred in 3 patients (12.0%), all in the LAMS-only group (0% vs. 16.7%, p = 0.641). Mean stent dwell time was shorter in the stricturoplasty group (52.6 vs. 90.4 days, p = 0.896), and the stricturoplasty group had significantly higher rates of prior ≥ 3 balloon dilations (85.7% vs. 16.7%, p = 0.006), suggesting a more refractory phenotype.

**Conclusions:**

LAMS is a safe and effective treatment for GJ strictures after RYGB, with high short-term success. In patients with multiple prior dilations or complex fibrotic strictures, concurrent stricturoplasty appears to be a technically feasible adjunct that may reduce stent migration and improve durability. These trends support further investigation.

Management of recalcitrant gastrojejunal (GJ) anastomotic strictures remains a significant challenge following Roux-en-Y gastric bypass (RYGB), often requiring repeated interventions and, in some cases, surgical revision. Occurring in up to 0.3–3% of patients within first year after surgery, these strictures arise from ischemia, marginal ulceration, or chronic mucosal inflammation [1, 2] and manifest clinically as progressive dysphagia, postprandial pain, nausea, vomiting, or weight loss, significantly impairing quality of life [3]. While endoscopic balloon dilation is effective and remains the first-line therapy, its long-term success may be limited, with variable durable symptom relief and some requiring multiple sessions or ultimately progressing to surgical revision [3, 4]. Surgical revision, while definitive, carries operative risk and technical difficulty in the post-bariatric anatomy. Given the relative morbidity associated with revision and sometimes variable efficacy of repeat dilation, alternative endoscopic strategies are increasingly being explored.

Lumen-apposing metal stents (LAMS) have emerged as a promising intervention for refractory GJ strictures, offering technical ease, wider luminal patency, and longer duration of radial expansion [5]. Several studies have demonstrated favorable short-term outcomes with LAMS, but questions remain regarding long-term durability, stent migration, and lack of mucosal remodeling [6]. In clinical practice, LAMS alone may not be sufficient in patients with dense fibrotic or recurrent strictures [7]. However, important knowledge gaps remain regarding which patient will benefit most from LAMS, the optimal timing of LAMS deployment relative to dilation failure and mucosal healing, and the long-term durability of LAMS therapy in maintaining symptom resolution and avoiding reintervention-. We evaluated a novel approach that combines LAMS placement with concurrent endoscopic stricturoplasty. While endoscopic stricturoplasty has been described for non-anastomotic GI strictures, its application at the GJ anastomosis has not been widely reported.

This study represents the first investigation of this combined technique in patients with RYGB-related GJ strictures, particularly those with a history of multiple failed dilations or known risk factors such as nonsteroidal anti-inflammatory drug (NSAID) use. We hypothesized that the addition of stricturoplasty may enhance the durability of endoscopic management in this patient population versus conventional endoscopic dilation strategies.

## Methods

We conducted a single-center, retrospective cohort study at Cleveland Clinic including consecutive patients treated between January 1, 2020, and May 31, 2025. Patients underwent intervention as determined by their treatment team and were assigned a priori to two exposure cohorts based on the index endoscopic intervention: (1) LAMS only and (2) LAMS with concurrent endoscopic stricturoplasty.

The study was approved by the Cleveland Clinic Foundation Institutional Review Board (IRB 15–227) with a waiver of informed consent due to minimal risk and use of de-identified data. All procedures described in this study were performed as part of routine clinical care at the discretion of the treating endoscopist. The use of concurrent endoscopic stricturoplasty was not performed under a prospective investigational protocol, but rather as an individualized therapeutic decision based on clinical judgment, endoscopic findings, and prior treatment history. No patients were enrolled or treated under a research protocol for this intervention.

Inclusion criteria were: adults (≥ 18 years) with prior metabolic surgery involving a GJ anastomosis who developed a GJ stricture and underwent endoscopic management with a LAMS. Exclusion criteria were non-GJ strictures, malignant or inflammatory bowel disease related strictures, use of non-LAMS stents as the index device, or insufficient documentation precluding outcome ascertainment.

Data were collected from the electronic health record using a standardized form. Collected variables included demographic factors including age, sex, and body mass index (BMI) at the time of LAMS placement; the date of primary metabolic surgery (or the first operation involving a GJ anastomosis); and comorbidities (diabetes mellitus, hypertension, cardiovascular disease, and chronic kidney disease). Medication use, including biologic or other immunosuppressive therapies, was reviewed through detailed chart abstraction. No patients in the cohort were receiving biologic or immunosuppressive medications at the time of LAMS placement. Risk factors collected were tobacco use, NSAID use, and Helicobacter pylori (HP) status. Stricture and procedure-related variables comprised the date of GJ stricture diagnosis, the date of LAMS placement, procedure duration (minutes), stent characteristics (size), whether concurrent stricturoplasty was performed (yes/no), and the number of prior balloon dilations categorized as ≥ 3 or not. Time to event measures were defined a priori as follows: dwell time, the interval from LAMS placement to stent removal; stricture to procedure interval, defined as the time from the documented GJ stricture diagnosis to index LAMS placement (not prior dilation procedures); and follow-up duration, the interval from LAMS placement to the last recorded clinic or procedural encounter. Adverse events included stent migration, defined as endoscopic or radiographic displacement from the intended position, perforation, bleeding, and any other events documented in the endoscopy or clinical notes.

Clinical success was defined as adequate tolerance of the stent with partial or complete resolution of presenting symptoms. We prespecified short-term success as occurring within ≤ 30 days, and long-term success as sustained beyond > 90 days, without need for unplanned re-intervention.

All procedures were performed by experienced therapeutic endoscopists under standard anesthesia and peri-procedural care. LAMS devices were deployed across the GJ anastomotic narrowing under endoscopic guidance. When performed, concurrent endoscopic stricturoplasty was performed using a standardized four-quadrant radial incision technique with an IT2 knife (Olympus ITknife2™) to release the fibrotic ring. This approach is conceptually consistent with previously described endoscopic stricturotomy techniques for benign gastrointestinal and anastomotic strictures, in which radial incisions using needle-knife or insulated-tip devices are used to disrupt fibrotic tissue and improve luminal patency [8, 9].

Choice of LAMS alone versus LAMS with concurrent stricturoplasty was non-random and not protocolized. Decisions were made by the treating endoscopist at the time of the index procedure based on the endoscopic impression of fibrosis, degree and distensibility of the anastomotic lumen, history of prior dilations, perceived migration risk, and practical considerations. These pragmatic factors were not standardized or adjudicated. To partially characterize selection, we abstracted prespecified proxies (numbers of previous ballon dilation) and report baseline imbalances in the Results.

The primary outcome was long-term clinical success (> 90 days) as defined previously. Secondary outcomes included: short-term clinical success (≤ 30 days), stent migration, procedure duration, dwell time, follow-up duration, need for re-intervention (repeat dilation, re-stenting, or surgical revision), and other adverse events.

Continuous variables are summarized as mean ± standard deviation (SD) and median [interquartile range, IQR]; categorical variables are presented as counts (percentages). Between-cohort comparisons for continuous variables were performed using two-sided independent-samples t tests. Statistical significance was defined as α = 0.05. Analyses were conducted on a complete case basis for each outcome. Records lacking a stent removal date were excluded from dwell-time analyses; patients without a subsequent clinic or procedural note were censored at their last documented encounter for follow-up duration. No imputation was performed. Analyses were performed using SPSS Statistics, version 28.0 (IBM Corp., Armonk, NY, USA).

## Results

A total of 25 patients who underwent LAMS placement for GJ strictures following metabolic surgery were included in the analysis. The most common presenting symptoms included dysphagia, nausea, vomiting, and intolerance to oral intake, consistent with typical manifestations of GJ strictures. Of these, 7 patients (28.0%) underwent concurrent stricturoplasty at the time of LAMS placement, while 18 patients (72.0%) received LAMS alone.

The mean age of the overall cohort was 46.8 ± 12.9 years, with a median of 51.9 years (IQR: 39.1–56.8). The age distribution was comparable between groups: 47.3 ± 14.6 years (median 48.1, IQR: 36.9–57.4) in the stricturoplasty group and 46.6 ± 12.9 years (median 52.0, IQR: 39.2–55.3) in the non-stricturoplasty group (p = 0.022). Time from index metabolic surgery to GJ stricture diagnosis was notably shorter in the stricturoplasty group, with a mean duration of 4.1 ± 7.3 years (median 1.4 years, IQR: 0.3–1.6), compared to 10.0 ± 8.0 years (median 11.4 years, IQR: 2.7–16.9) in the non-stricturoplasty group (p = 0.692).

In the total patient cohort, 19 (76.0%) were female, with similar proportions observed between groups (5/7, 71.4% vs. 14/18, 77.8%; p = 1.000). Diabetes mellitus was present in 8 patients (32.0%), including 2/7 (28.6%) in the stricturoplasty group and 6/18 (33.3%) in the non-stricturoplasty group (p = 1.000). Hypertension was reported in 12 patients (48.0%), distributed evenly across groups (4/7, 57.1% vs. 8/18, 44.4%; p = 0.900). Cardiovascular disease was identified in 2 patients (8.0%), both of whom were in the non-stricturoplasty group (0/7 vs. 2/18; p = 0.922). Chronic kidney disease was noted in 3 patients (12.0%), with 1 case in the stricturoplasty group and 2 in the non-stricturoplasty group (14.3% vs. 11.1%, p = 1.000).

Smoking history was reported in 15 patients (60.0%), including 4/7 (57.1%) in the stricturoplasty group and 11/18 (61.1%) in the non-stricturoplasty group (p = 1.000). NSAID use was observed exclusively in the stricturoplasty group, with 4/7 patients (57.1%) reporting recent or chronic use, while none of the patients in the non-stricturoplasty group reported NSAID use (0/18, p = 0.004). HP infection was observed in only 2 patients (8.0%), both of whom were in the non-stricturoplasty group (0/7 vs. 2/18; p = 0.922). Finally, prior balloon dilation of ≥ 3 sessions was noted in 9 patients (36.0%), with a markedly higher rate in the stricturoplasty group (6/7, 85.7%) compared to the non-stricturoplasty group (3/18, 16.7%; p = 0.006), reflecting the more treatment-refractory nature of strictures in these patients. Full details in Table 1.

**Table 1:** Characteristics of patients underwent Lumen-apposing metal stents

Variable	Total cohort (n, %)	Stricturoplasty (n = 7)	LAMS alone (n = 18)	P value
Age	46.8 ± 12.9	47.3 ± 14.6	46.6 ± 12.9	0.022
	51.9 (39.1, 56.8)	48.1 (36.9, 57.4)	52.0 (39.2, 55.3)	
Surgery to stricture (years)	8.7 ± 8.1	4.1 ± 7.3	10.0 ± 8.0	0.692
	8.5 (1.2, 15.1)	1.4 (0.3, 1.6)	11.4 (2.7, 16.9)	
Female	19 (76.0%)	5 (71.40%)	14 (77.80%)	1
DM	8 (32.0%)	2 (28.60%)	6 (33.30%)	1
HTN	12 (48.0%)	4 (57.10%)	8 (44.40%)	0.900
CVD	2 (8.0%)	0 (0.00%)	2 (11.10%)	0.922
CKD	2 (12.0%)	1 (14.30%)	2 (11.10%)	1
Smoking	15 (60.0%)	4 (57.10%)	11 (61.10%)	1
NSAIDs use	4 (16.0%)	4 (57.10%)	0 (0.00%)	0.004
HP +	2 (8.0%)	0 (0.00%)	2 (11.10%)	0.922
Balloon dilation > 3 +	9 (36.0%)	6 (85.50%)	3 (16.70%)	0.006

*DM* diabetes mellitus, *HTN* hypertension, *CVD* cardiovascular disease, *CKD* chronic kidney disease, *NSAID* non-steroid anti-inflammation drug, *HP* helicobacter pylori, *LAMS* lumen-apposing metal stents

### Procedure outcomes

Short-term clinical success was achieved in 22 of 25 patients (88.0%) following LAMS placement. Success rates were comparable between the stricturoplasty group (6/7, 85.7%) and the LAMS-only group (16/18, 88.9%; p = 1.000). Long-term clinical success was maintained in 16 patients (64.0%), 5 of 7 (71.4%) in the stricturoplasty group and 11 of 18 (61.1%) in the LAMS-only group (p = 0.985).

Two patients in the stricturoplasty group did not achieve long-term clinical success. One patient had a prior history of perforated marginal ulcer requiring surgical intervention before undergoing LAMS placement and stricturoplasty. This patient continued to have persistent symptoms despite treatment and ultimately underwent GJ revision. The other patient underwent re-stenting due to persistent symptoms but continued to experience abdominal pain. In the LAMS-only group, two patients required GJ revision—one for symptom worsening and one for stent obstruction. An additional three patients in this group experienced symptom recurrence following initial improvement, including one who underwent redo GJ and another who had the stent removed due to pain. In total, 7 of 18 patients (38.9%) in the LAMS-only group failed to achieve durable long-term success.

The mean stent dwell time across the cohort was 78.4 ± 54.8 days, with no statistically significant difference observed in the stricturoplasty group (52.6 ± 17.6 days) compared to the LAMS-only group (90.4 ± 62.4 days), (p = 0.896). The median dwell time was 57.0 days (IQR: 46.0–99.0) overall, with subgroup medians of 49.0 days (IQR: 43.0–51.5) and 91.0 days (IQR: 50.0–128.5), respectively. Similarly, the mean procedure time was slightly longer in the stricturoplasty group (24.5 ± 9.9 min) versus the LAMS-only group (18.7 ± 12.7 min), with a median of 16.0 min (IQR: 11.0–30.0) across the entire cohort. Median follow-up duration was 280 days (IQR: 112–372), with a longer interval observed in the LAMS-only group (312 days, IQR: 209–361) compared to the stricturoplasty group (170 days, IQR: 103–364), though without statistical significance. The interval between stricture diagnosis and LAMS placement was also similar between groups (531.3 ± 954.2 days overall; 625.1 ± 1430.5 vs. 494.8 ± 747.0; p = 0.545). Stent migration occurred in 3 patients (12.0%), all from the LAMS-only group (0/7 vs. 3/18; p = 0.641), and none of these cases were symptomatic or required further intervention. In terms of LAMS size, the most used configuration was 10 mm × 15 mm (13/25, 52.0%), followed by 10 mm × 20 mm (9/25, 36.0%) and 15 mm × 15 mm (2/25, 8.0%). The stricturoplasty group predominantly received the 10 × 15 mm stent (6/7, 85.7%), whereas the LAMS-only group had more variation in stent sizing. Full details in Table 2.

**Table 2:** Outcomes of patients underwent Lumen-apposing metal stents

Outcome	Total cohort (n, %)	Stricturoplasty (n = 7)	LAMS alone (n = 18)	P-value
Dwell time	78.36 ± 54.84	52.57 ± 17.64	90.40 ± 62.37	0.896
	57.0 (46.0, 99.0)	49.0 (43.0, 51.5)	91.0 (50.0, 128.5)	
Short term (≤ 30 Days)	22 (88.0%)	6 (85.5%)	16 (88.9%)	1
Long term (> 90 days)	16 (64.0%)	5 (71.4%)	11 (61.1%)	0.985
Stent migration	3 (12.0%)	0 (0%)	3 (16.7%)	0.641
Procedure time	20.33 ± 12.05	24.50 ± 9.87	18.67 ± 12.73	
	16.00 (11.00–30.00)	23.00 (16.00–33.00)	13.00 (10.50–26.00)	
Follow up (days)	273.29 ± 180.44	289.86 ± 278.04	266.47 ± 132.84	
	280 (112, 372)	170 (103, 364)	312 (209, 361)	
Stricture-to-procedure interval (days)	531.3 ± 954.2	625.1 ± 1430.5	494.8 ± 747.0	0.545
	94.0 days (60.0–331.0)	69.0 days (48.5–166.5)	144.0 days (60.8–440.5)	
GJ revision	4 (16%)	1 (14.3%)	3 (16.7%)	1
LAMS size				
10*15 mm	13	6	7	
15*15 mm	2	0	2	
10*20 mm	9	1	8	
Unknown	1	0	1	

*LAMS* lumen-apposing metal stents, *GJ* gastrojejunal

## Discussion

In this retrospective study, we evaluated the use of LAMS for the management of GJ strictures following RYGB and explored the feasibility of combining LAMS placement with concurrent endoscopic stricturoplasty. Overall, LAMS and concurrent stricturoplasty were associated with both favorable short- and long-term clinical success rates (85.5% and 71.4% respectively). To our knowledge, this is the first study to report the use of stricturoplasty performed at the time of LAMS deployment for GJ strictures after RYGB. In patients with refractory disease—often with multiple prior dilations or known risk factors—this combined approach appeared technically feasible, safe, and durable.

Our findings align with and extend the growing literature on endoscopic management of refractory GJ strictures [10–13]. Balloon dilation remains the initial therapy for most GJ strictures; with reported long-term success rates variable and a some proportion of patients requiring multiple sessions or ultimately proceeding to surgical revision [3, 14]. For patients who fail conventional dilation, LAMS offers a minimally invasive alternative that provides durable luminal patency through its anchoring flanges and wide-diameter conduit.

Several prior studies have reported favorable outcomes with LAMS alone in the treatment of GJ strictures, with short-term clinical success typically between 75 and 90%, and variable long-term durability depending on patient selection and follow-up [12, 13]. Stent migration remains a known complication, with rates reported up to 10% in some series [6, 12]. Our cohort demonstrated a comparable migration rate (12.0%); importantly, all instances were asymptomatic, with no cases of clinically significant obstruction, and did not require reintervention, consistent with the generally favorable safety profile of LAMS in this population. The present findings support the growing evidence base for LAMS as a viable rescue strategy for refractory GJ strictures and extend its potential applicability when combined with adjunctive techniques like stricturoplasty.

While our study was underpowered to detect statistically significant differences in outcomes, several observations suggest that stricturoplasty may offer advantages in selected patients. The technique involves incising the fibrotic stricture, which may increase the risk of bleeding, yet in our cohort, procedural safety was preserved with no major complications reported. Importantly, no cases of stent migration occurred in the stricturoplasty group, compared to 12% (n = 3) in the LAMS-only group. Although not conclusive, this trend raises the possibility that stricturoplasty may reduce migration risk by improving stent tissue purchase or anchoring. In our clinical practice, endoscopic balloon dilation remains the first-line therapy for GJ strictures, and patients typically undergo two to three dilation sessions prior to consideration of LAMS placement if symptoms persist. While other endoscopic approaches have been described, these are not routinely incorporated into our treatment pathway. The decision to perform concurrent stricturoplasty is not standardized and is made on a case-by-case basis at the discretion of the endoscopist. In this study, patients selected for stricturoplasty more frequently had features suggestive of a refractory phenotype, including multiple prior dilations and fibrotic-appearing strictures. In contrast, general comorbidities such as hypertension and cardiovascular disease were not expected to directly influence stricture behavior or response to endoscopic therapy and are presented primarily for baseline cohort characterization. These findings may help generate hypotheses regarding patient selection for concurrent stricturoplasty such as in cases with dense fibrous tissue or anatomic features predisposing to stent migration. While preliminary, this approach could serve as a starting point for future algorithm development, pending validation in prospective, multicenter studies.

Our findings offer preliminary insights that may help inform future investigation and hypothesis generation in the management of GJ strictures. When using LAMS to manage GJ strictures, clinicians must weigh the potential benefits of concurrent stricturoplasty—particularly the observed trend toward reduced stent migration against the theoretical risk of bleeding associated with mucosal incision. Notably, no clinically significant bleeding events were observed in our cohort, suggesting that the procedure may be safe in appropriately selected patients. While our data do not demonstrate a clear improvement in long-term durability or clinical success, stricturoplasty may be considered in select cases where stent migration is a concern. In such patients, early consideration of concurrent stricturoplasty may help improve long-term outcomes while avoiding surgical revision. Given the technical demands of both LAMS deployment and endoscopic stricturoplasty, these procedures should be performed by experienced endoscopists familiar with advanced tissue manipulation techniques to avoid adverse events [15]. Importantly, while stent migration occurred in a minority of patients (12.0%), all instances were asymptomatic and did not require reintervention, reinforcing the overall safety of this approach when performed in a controlled setting.

This study has several limitations. First, its retrospective design and the small sample size, particularly within the stricturoplasty subgroup, limit the statistical power and generalizability of our findings. Second, there was no standardized algorithm guiding the decision to perform stricturoplasty, introducing potential selection bias in the choice of combined therapy, which may be biased to more difficult or fibrotic strictures. Lastly, we were unable to control for the underlying etiology of the GJ stricture, and how the GJ was performed at original surgery, which may independently influence treatment response and outcomes.

Despite these limitations, our findings suggest that concurrent stricturoplasty is a feasible and safe adjunct to LAMS in carefully selected patients. While the difference did not reach statistical significance, we observed a numerically higher rate of long-term clinical success and a notable reduction in stent migration with concurrent stricturoplasty compared to LAMS alone. These findings support the potential utility of stricturoplasty in select patients and underscore the need for larger, prospective studies to validate these early observations. Future prospective, multicenter studies with larger cohorts, standardized treatment protocols, and inclusion of patient-reported outcomes are warranted to validate these findings and better define optimal patient selection.

## Conclusion

LAMS is a safe and effective option for managing GJ strictures after RYGB, particularly in patients who fail conventional balloon dilation. Our preliminary experience suggests that concurrent stricturoplasty is technically feasible and well tolerated in select patients with refractory strictures. While our study was not powered to demonstrate definitive improvements in long-term clinical success, we observed a notable trend toward reduced stent migration in the stricturoplasty group. This finding may support the use of stricturoplasty as an adjunctive strategy in anatomies at high risk for migration. Further prospective, multicenter studies are needed to validate these observations and to establish standardized criteria for incorporating stricturoplasty into the endoscopic management algorithm for GJ strictures.
